# Removal of antimony from model solutions, mine effluent, and textile industry wastewater with Mg-rich mineral adsorbents

**DOI:** 10.1007/s11356-022-23076-8

**Published:** 2022-09-23

**Authors:** Hanna Runtti, Tero Luukkonen, Sari Tuomikoski, Tao Hu, Ulla Lassi, Teija Kangas

**Affiliations:** 1 Research Unit of Sustainable Chemistry, University of Oulu, P.O. Box 4300, FI-90014 Oulu, Finland; 2 Fibre and Particle Engineering Research Unit, University of Oulu, P.O. Box 8000, FI-90014 Oulu, Finland

**Keywords:** Adsorption, Anion exchange, Antimony, Brucite, Hydromagnesite, Layered double hydroxides

## Abstract

Naturally occurring layered double hydroxide mineral, brucite (BRU), was compared with hydromagnesite (HYD) and a commercial Mg-rich mineral adsorbent (trade name AQM PalPower M10) to remove antimony (Sb) from synthetic and real wastewaters. The BRU and HYD samples were calcined prior to the experiments. The adsorbents were characterized using X-ray diffraction, X-ray fluorescence, and Fourier transform infrared spectroscopy. Batch adsorption experiments were performed to evaluate the effect of initial pH, Sb concentration, adsorbent dosage, and contact time on Sb removal from synthetic wastewater, mine effluent, and textile industry wastewater. Several isotherm models were applied to describe the experimental results. The Sips model provided the best correlation for the BRU and M10. As for the HYD, three models (Langmuir, Sips, and Redlich–Peterson) fit well to the experimental results. The results showed that the adsorption process in all cases followed the pseudo-second-order kinetics. Overall, the most efficient adsorbent was the BRU, which demonstrated slightly higher experimental maximum adsorption capacity (27.6 mg g^-1^) than the HYD (27.0 mg g^-1^) or M10 (21.3 mg g^-1^) in the batch experiments. Furthermore, the BRU demonstrated also an efficient performance in the continuous removal of Sb from mine effluent in the column mode. Regeneration of adsorbents was found to be more effective under acidic conditions than under alkaline conditions.

## Introduction

Antimony (Sb) is a metalloid element that exists in the environment mainly as Sb(III) or Sb(V). The common Sb-containing minerals include stibnite (Sb_2_S_3_) and valentinite (Sb_2_O_3_), which are frequently associated with copper, silver, or lead ores, coal, and petroleum (Filella et al. 2002). Correspondingly, mining and smelting activities and burning of fossil fuels are the major emission sources of Sb (Ungureanu et al. 2015). Commercially, Sb is used in flame retardants, in paint pigments, as additive in ceramics, glass, or lead, and as semiconductor (Filella et al. 2002). In aqueous environments, Sb is present as soluble hydroxides (e.g, Sb(OH)_6_^−^) or it can form complexes with chloride, fluoride, or bromide (Schweitzer and Pesterfield 2010). The aqueous concentrations of Sb range from < 1 μg L^−1^ in uncontaminated natural waters to few mg L^−1^ in mine effluents and further to > 1 g L^−1^ in geothermal waters (Filella et al. 2002; Flaková et al. 2017). Sb is toxic or even lethal at elevated intake levels, but there is no conclusive evidence indicating that prolonged oral exposure to low Sb concentrations would exert carcinogenic or mutagenic effects in humans (International Agency for Research on Cancer (IARC) 1989; World Health Organization (WHO), 2003). Nevertheless, the safe Sb levels in drinking water were set at 5, 6, and 20 μg L^−1^ by the European Union (European Council 1998), by the United States Environmental Protection Agency ( 2009), and by the WHO ( 2011), respectively. In addition, Sb is on the European Union list of critical raw materials (European Commission 2020).

Sb removal from contaminated waters has been studied using chemical precipitation (Enders and Jekel 1994b; Multani et al. 2017; Parker et al. 1979), coagulation–flocculation (Djuvfelt 2014; Enders and Jekel 1994a; Guo et al. 2009), biological treatment (Sun et al. 2016; Zhang et al. 2016), ion exchange (Parker et al. 1979), or reverse osmosis (Kang et al. 2000). Adsorption has also received considerable attention (Cheng et al. 2022; Dong et al. 2022; Lee et al. 2018a, 2018b; Ungureanu et al. 2015; Xie et al. 2022; Yan et al. 2022; Zhang et al. 2019). Oxidation of Sb(III) prior to treatment is beneficial, as Sb(V) is generally more efficiently removed and less toxic. However, many conventional water treatment processes are ineffective for Sb removal, or they can be prohibitively expensive.

One potential group of adsorbents for Sb removal are layered double hydroxides (LDHs), which can exchange their interlayer anions for Sb(OH)_4_^−^. Sb adsorption has already been studied using synthetic LDHs: Zn-Al sulfate (Ardau et al. 2016), Zn-Fe (Lu et al. 2015), Fe-Al (Kameda et al. 2009), Mg-Al (Kameda et al. 2015), Cu-Al (Kameda et al. 2012), and magnetic nanoparticles that support calcined LDHs (Lee et al. 2018b). LDHs are also available as naturally occurring minerals, such as brucite (BRU).

This study investigates the use of the BRU and hydromagnesite (HYD), which is commonly associated with weathering products of BRU, for aqueous Sb removal and compare their individual performances with that of a commercial magnesium-rich mineral adsorbent (trade name AQM PalPower M10). The experimental results were also compared with the reported findings obtained using synthetic LDHs. M10 consists mainly of magnesium, iron, and silicon-rich phases, and it has been applied in passive treatment systems for the removal of metals (Zn, Ni, Cd, Cu, and Pb) from urban runoff waters (Gogoi et al. 2018) or mine effluents (Postila et al. 2019). The research questions of this study are as follows: (1) Is naturally occurring LDH BRU suitable for the treatment of synthetic and real waters containing Sb? (2) How does the efficiency of natural LDHs compare with that of synthetic LDHs? (3) How stable are Sb-laden LDHs and what is their reusability potential? The motivation behind this study is that naturally occurring LDHs are a potentially cost effective and readily available alternatives to synthetic LDHs. In this study, batch adsorption experiments involving synthetic solutions, real mine effluent, and textile wastewaters were performed to evaluate the influence of initial pH, Sb concentration, adsorbent dosage, and contact time on Sb removal. Sb desorption was tested under acidic and alkaline conditions to evaluate the stability of the spent adsorbents and assess their regenerability. Moreover, several isotherm and kinetic models were applied to the obtained data. The preliminary suitability of the adsorbents for the treatment of Sb-laden mine effluent was determined through batch and column experiments.

## Materials and methods

### Materials

The BRU, HYD, and M10 were obtained from a Finnish supplier. The M10 was used as received, whereas the BRU and HYD were calcined at 500 °C and 700 °C, respectively, washed with deionized water, and then dried at 105 °C. Particle sizes of 63–125 μm were separated and used in the batch experiments. For the column experiments, BRU particles of 0.5–1 mm were used.

Synthetic wastewater was prepared by dissolving antimony trichloride (SbCl_3_) (99%, Alfa Aesar) in ultrapure water. A stock solution of Sb(III) (25 mg L^−1^) was first prepared by adding sodium hydroxide (NaOH) (~1.4 g L^−1^) to facilitate the dissolution of SbCl_3_. Then, the stock solution was diluted to the required concentrations. The pH was adjusted using 1.0 or 0.1 M hydrochloric acid (HCl) and NaOH (FF-Chemicals).

The real wastewater samples were mine effluent and textile industry wastewater. The mine effluent (settled drainage water treated with ferric sulfate) was obtained from an underground gold mine in Finland. For the textile industry wastewater, the source of Sb was the flame retardants used in textiles. The compositions of the wastewater samples are presented in Table 1.

**Table 1:** The compositions of the wastewater samples

Compound	Concentration [mg L^−1^] in mine effluent^a^	Concentration [mg L^−1^] in textile wastewater^a^
NH_4_^+^	2.9	-
Al	<0.03	0.19
As	<0.015	<0.015
B	0.080	<0.02
Ba	0.040	0.14
Be	<0.005	<0.005
Ca	315	3.00
Cd	<0.002	<0.002
Co	<0.003	<0.003
Cr	<0.01	0.046
Cu	<0.005	0.010
Fe	0.030	0.17
K	9.75	23.6
Mg	101	1.60
Mn	0.99	0.022
Mo	0.010	<0.005
Na	54.9	3950
Ni	0.10	0.011
P	<0.05	24.4
Pb	<0.015	<0.015
S	382	1840
Sb	0.20	21.4
Se	-	<0.015
Sn	-	<0.015
Ti	-	<0.015
V	-	0.008
Zn	-	1.29

^a^Determined using an optical emission spectrometer (metals) or flow analysis (CFA and FIA) and spectrometric detection (ammonium nitrogen) by an accredited laboratory

### Characterization methods

X-ray diffraction (XRD) and X-ray fluorescence (XRF) analyses were conducted to confirm the mineralogy and chemical composition of the adsorbents. The XRD patterns were recorded by a PANalytical X’Pert Pro X-ray diffractometer (Malvern Panalytical) using monochromatic CuKα1 radiation (*λ*=1.5406 Å) at 45 kV and 40 mA. Diffractograms were collected within the 2θ range 10°–90° at 0.017° intervals and with a scan step time of 100 s. The crystalline phases and structures of the adsorbents were analyzed using the HighScore Plus software (Version 4.0, PANalytical). The peaks were identified according to the International Centre for Diffraction Data (ICDD) (PDF-4+ 2020 RDB). The phases were quantified through Rietveld analysis using HighScore. XRF spectra were recorded by a PANalytical Axios mAX XRF spectrometer, wherein the samples were prepared as loose powders using a mylar film under helium atmosphere at 4 kW.

The Fourier transform infrared spectroscopy (FTIR) spectra of the adsorbents were collected using a Perkin Elmer Spectrum One spectrometer equipped with an attenuated total reflectance unit.

### Batch adsorption experiments

Batch adsorption experiments were conducted to determine the effects of initial pH, Sb concentration, adsorbent dosage, and contact time on Sb removal efficiency. All adsorption experiments, except the kinetic studies, were performed in 50 mL centrifuge tubes (25 mL water volume) placed on a shaker. The kinetic studies were performed in a 1-L reactor vessel (800 mL water volume) equipped with a magnetic stirrer and subjected to an agitation speed of 1000 rpm. All adsorption experiments were performed in duplicate. The parameters for each experiment are shown in Table 2.

**Table 2:** Parameters for the batch experiments conducted to determine the effects of initial pH, Sb concentration, adsorbent dosage, and contact time on Sb removal in synthetic solutions, mine effluent, and textile wastewater. All experiments were conducted at room temperature (~22–23 °C)

Parameters	Initial pH	C_0_ [mg L^−1^]	Adsorbent dosage [g L^−1^]	Contact time [min h^−1^]	Water type
Initial pH	2.0–10.0	25.0	5.0	24 h	Synthetic
Adsorbent dosage	4.0	18.0	0.5–5.0	24 h	Synthetic
	7.2	0.2	0.1–15	24 h	Mine effluent
	7.9	22.1	0.1–15	24 h	Textile wastewater
Sb concentration	4.0	5.0–25.0	0.5	24 h	Synthetic
Contact time	4.0	13.0	0.5	1 min–48 h	Synthetic
	7.2	0.2	5.0	2 min–24 h	Mine effluent

After the desired contact time had elapsed, the samples were centrifuged (3500 rpm, 5–15 min), and the supernatant was collected using a pipette. The Sb concentration was measured with an optical emission spectrometer (Thermo Electron IRIS Intrepid II XDL Duo and Perkin Elmer Optima 5300 DV). The removal efficiencies (*R%*) and adsorption capacities *q*_*e*_ (mg g^−1^) of the adsorbents were calculated using the following equations:

1 $$R\%=\frac{C_0-{C}_e}{C_0}\times 100\%$$

and

2 $${q}_e=\frac{\left({C}_0-{C}_e\right)V}{m}$$

where *C*_*0*_ and *C*_*e*_ (mg L^−1^) are the initial and equilibrium liquid phase Sb ion concentrations, respectively, *V* (L) is the volume of the solution, and *m* (g) is the mass of the adsorbent.

#### Adsorption isotherms

Langmuir (1918), Freundlich (1906), Sips (1948), Bi-Langmuir (Graham 1953), Dubinin–Radushkevich (DR) (Dubinin and Radushkevich 1947), Redlich–Peterson (RP) (Redlich and Peterson 1959), and Temkin and Pyzhev (1940) isotherm models were applied to the experimental results for the Sb adsorption onto the BRU, HYD, and M10. The DR and Temkin models did not fit the results, and the Bi-Langmuir model was reduced to the Langmuir model in all cases. Therefore, only the results for the Langmuir, Freundlich, Sips, and RP models are reported. The isotherm parameters were obtained through non-linear regression using OriginPro 2018 (OriginLab Corporation). The non-linear form of the Langmuir’s equation is:

3 $${q}_e=\frac{b_L{q}_m{C}_e}{1+{b}_L{C}_e}$$

where *b*_*L*_ (L mg^−1^) is the parameter related to energy of adsorption, *q*_*m*_ (mg g^−1^) is the maximum adsorption capacity, and *C*_*e*_ (mg L^−1^) is the Sb concentration in the solution in equilibrium.

The Freundlich model non-linear form is as follows:

4 $${q}_e={K}_F{C}_e^{1/{n}_F}$$

where *K*_*F*_ ((mg g^− 1^)(mg L^− 1^)^− 1/nF^ ) is the Freundlich constant related to adsorption capacity, and *n*_*F*_ (dimensionless) is the Freundlich constant related to adsorption intensity.

The Sips isotherm combines the properties of the two models above and is expressed as:

5 $${q}_e=\frac{q_m{\left({b}_S{C}_e\right)}^{n_s}}{1+{\left({b}_S{C}_e\right)}^{n_s}}$$

where *b*_*S*_ (L mg^−1^) is a constant related to adsorption energy, and *n*_*s*_ is a dimensionless constant characterizing the heterogeneity of the system.

The RP isotherm is modified from the Langmuir and Freundlich models and is expressed as:

6 $${q}_e=\frac{K_R{C}_e}{1+{a}_R{C}_e^{\beta }}$$

where *K*_*R*_ (L g^−1^) and *a*_*R*_ (L mg^−1^)^-β^ are RP isotherm constants, and *β* (dimensionless) is an exponent.

The suitability of the isotherm equations was evaluated by comparing the residual root mean square errors (*RMSE*). A small error function value indicates good curve fitting. An error function is defined as follows:

7 $$RMSE=\sqrt{\sum\nolimits_{i=1}^n{\left({q}_{e\left(\exp \right)}-{q}_{e(calc)}\right)}^2}$$

where *n* is the number of experimental data points, *p* is the number of parameters in the isotherm model, *q*_*e(exp)*_ (mg g^−1^) is the experimental adsorption capacity in equilibrium, and *q*_*e(alc)*_ (mg g^−1^) is the calculated adsorption capacity in equilibrium.

#### Kinetics of adsorption

The kinetic parameters of the adsorption experiments were solved using the non-linear forms of the pseudo-first-order (Lagergren 1898), pseudo-second-order (Ho and McKay 1999), and Elovich (Zeldowitsch 1934) models. These models are typically applied to adsorption processes. OriginPro 2018 was used to obtain the kinetic model parameters.

The pseudo-first-order rate is expressed as follows:

8 $${q}_t={q}_e\left(1-{e}^{-{k}_ft}\right)$$

where *q*_*e*_ (mg g^−1^) is the amount of adsorbed Sb at equilibrium, *q*_*t*_ (mg g^−1^) is the amount of adsorbed Sb at time *t* (min), and *k*_*f*_ (min^−1^) is the pseudo-first-order rate constant.

The pseudo-second-order process is expressed as follows:

9 $${q}_t=\frac{{q_e}^2{k}_st}{q_e{k}_st+1}$$

where *k*_*s*_ (g mg^−1^ min^−1^) is the rate constant of the pseudo-second-order kinetics.

The Elovich equation can be written as follows:

10 $$\mathrm{q}=\frac{1}{\beta}\ln \left({\upsilon}_0\beta +\frac{1}{\beta } lnt\right)$$

where *υ*_0_ (mg g^−1^ min^−1^) is the initial adsorption rate, and *β* (g mg^−1^) is the desorption constant.

The suitability of the kinetic models was compared based on the RMSE value in Eq. 7.

#### Intra-particle diffusion model

In identifying the diffusion mechanism, the intra-particle diffusion model based on the theory proposed by Weber and Morris (1963) was used. The Weber−Morris equation is as follows:

11 $${q}_t={K}_{id}{t}^{1/2}+C$$

where *K*_*id*_ (mg g^−1^ h^−1/2^) is the intra-particle diffusion rate constant, and *C* is the intercept.

### Desorption experiments

The adsorbents were loaded with Sb by mixing 2.5 g of BRU, HYD, or M10 with 500 mL of 25 mg L^−1^ Sb solution for 2 h. Then, the adsorbents were collected through centrifugation, dried at 105 °C overnight, and kept in an exicator. The desorption experiments were conducted by transferring 0.5 g of the Sb-loaded adsorbents into 100 mL distilled water, 1 M HCl, or 1 M NaOH and then mixed for 2 h. Subsequently, the adsorbents were isolated using a centrifuge, and the Sb concentration in the aqueous solution was measured. Desorption efficiency (*%RE*) was calculated by using Eq. 12:

12 $$\%\mathrm{RE}=\frac{q_r\ }{q_0} \times 100\%$$

where *q*_*0*_ and *q*_*r*_ are the adsorption capacities of the adsorbents before and after regeneration, respectively.

### Column experiments

Column adsorption experiments were performed using only the most efficient adsorbent, that is, the BRU. A plastic column (diameter, 44.0 mm; height, 98.8 mm; volume, 0.15 L) was loaded with 100 g of BRU (particle size: 0.5−1 mm). The bed volume was approximately 90 mL. Mine effluent was pumped through the column by a peristaltic pump in the up-flow mode. The volumetric flow rates employed in the experiments were approximately 0.35, 0.18, and 0.09 L h^−1^ corresponding to 15 min, 30 min, and 1 h empty bed contact time. The empty bed contact time is determined as follows:

13 $$EBCT=\frac{V_m}{Q}$$

where *V*_*m*_ (L) is the bed volume, and *Q* (L h^−1^) is the volumetric flow rate. Samples were taken from the outlet of the column at different time intervals.

The cumulative amount of adsorbed Sb, *q*_total_ (mg) represents the area under the breakthrough curve at a given feeding concentration and flow rate. It is determined using the following equation:

14 $${q}_{\mathrm{total}}=Q{\int}_0^{\mathrm{total}}{C}_{ad} dt=Q{\int}_0^{\mathrm{total}}\left({C}_0-{C}_t\right) dt$$

where *C*_*0*_ (mg L^−1^) and *C*_*t*_ (mg L^−1^) are the influent and effluent concentrations, respectively, and *Q* (L h^−1^ is the flow rate through the column). The appropriate breakthrough time was chosen so that the effluent concentration from the column would be approximately 5% of the influent concentration (*C*_*0*_); also, the suitable exhaustion time was set so that the effluent concentration would be 95% of the influent concentration (*C*_*0*_).

The operation and dynamic response of the adsorption column were determined. The breakthrough and exhaustion times were used to evaluate the breakthrough curves.

The cumulative amount of Sb adsorbed per unit mass of adsorbent was calculated according to the following equation:

15 $${q}_e=\frac{q_{total}}{m},$$

where *m* (g) is the mass of the adsorbent.

## Results and discussion

### Characterization of the adsorbents

**Fig. 1:**
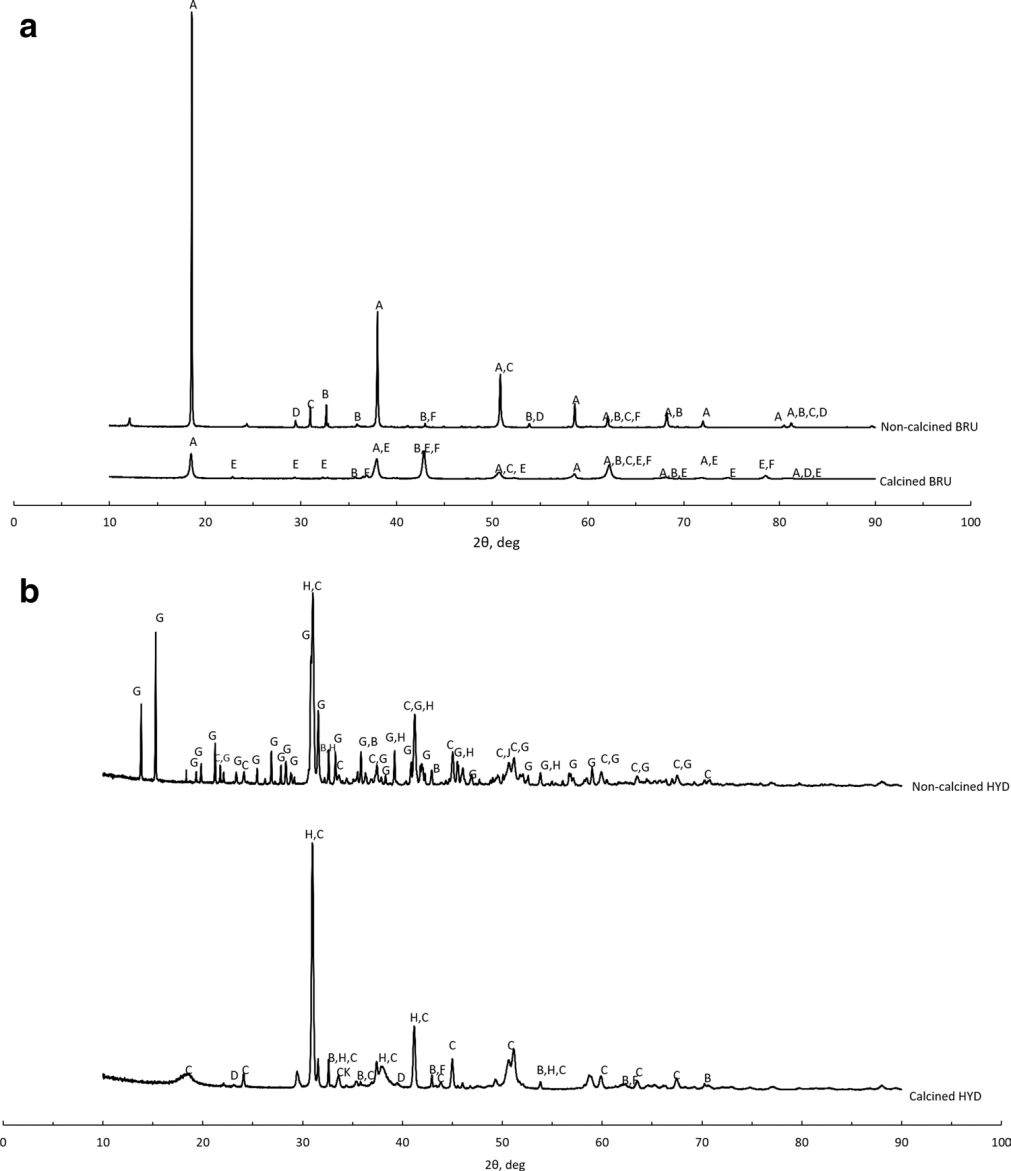
**a** X-ray diffraction patterns of the non-calcined and calcined BRU. A—Mg(OH)_2_ (04-011-5938), B—MgCO_3_ (04-009-2317), C—CaMg(CO_3_)_2_ (04-015-9838), D—CaCO_3_ (04-012-0489), E—Mg_2_SiO_4_ (01-084-1402), and F—MgO (04-005-4790). **b** X-ray diffraction patterns of the non-calcined and calcined HYD. B—MgCO_3_ (04-009-2317), C—CaMg(CO_3_)_2_ (04-015-9838), D—CaCO_3_ (04-012-0489), E—Mg_2_SiO_4_ (01-084-1402), F—MgO (04-005-4790), G—Mg_5_(CO_3_)_4_(OH)_2_(H_2_O)_4_ (mono: 04-013-7631, ortho: 04-012-8557), and H—CaMg_3_(CO_3_)_4_ (04-007-5228)

The chemical compositions of the HYD, BRU, and M10 are presented in Table 3. Expectedly, the adsorbents had a high magnesium content. The HYD contained a considerable amount of calcium, which is associated with dolomite and other carbonate phases (see Fig. 1). The adsorbents also contained some silicon, iron, sulfur, and potassium as minor elements.

**Table 3:** Main chemical constituents (normalized values) of the adsorbents as determined by XRF

Composition	Non-calcined BRU [w/w%]	Calcined BRU [w/w%]	Non-calcined HYD [w/w%]	Calcined HYD [w/w%]	M10 [w/w%]
MgO	83.6	87.7	56.3	58.0	51.7
SiO_2_	6.87	4.64	2.62	3.29	28.5
CaO	5.47	3.64	37.9	35.4	1.45
Fe_2_O_3_	0.764	0.783	0.23	0.196	12.1
SO_3_	0.078	0.273	0.038	0.038	1.90
K_2_O	0.028	0.042	0	0	0.052
NiO	0	0	0.024	0.019	0.302
MnO	0.078	0.093	0.012	0	0.256
Cr_2_O_3_	0.011	0	0.019	0	0.296
P_2_O_5_	1.42	1.12	1.246	0.89	0.621
Al_2_O_3_	0.364	0.372	0.117	0.175	1.49
SrO	0	0	0.268	0.224	0
Others^a^	0.065	0.056	0.055	0.53	0.104

^a^Including TiO_2_, Cl, As_2_O_3_, Co_3_O_4_, ZnO, and Na_2_O

The XRD patterns of the BRU and HYD are shown in Fig. 1a and 1b. Brucite (Mg(OH)_2_, ICDD 04-011-5938) was the main mineral phase in the BRU, and its contents were 91.2 and 51.8 weight-% in the non-calcined BRU and calcined BRU (Fig. 1a), respectively. The non-calcined BRU also contained magnesite (MgCO_3_, 04-008-0479, 5.0 weight-%), dolomite (CaMg(CO_3_)_2_, 00-036-0426, 2.7 weight-%), and calcite (CaCO_3_, 04-012-0489, 1.1 weight-%). As for the calcined BRU, the mineral phases were periclase (MgO, 04-005-4790, 42.1 weight-%), forsterite (Mg_2_SiO_4_, 01-084-1402, 6.0 weight-%), and calcite (04-012-0489, 0.1 weight-%).

In the non-calcined HYD (Fig. 1b), the main components were dolomite (CaMg(CO_3_)_2_, 42.7 weight-%) and hydromagnesite (Mg_5_(CO_3_)_4_(OH)_2_(H_2_O)_4_, 44.1 weight-%), which is a weathering product of brucite (Lechat et al. 2016). Moreover, it may contain both monoclinic (04-013-7631) and orthorhombic (04-012-8557) structures of hydromagnesite. It also contained 10.1 weight-% of huntite (CaMg_3_(CO_3_)_4_, 04-007-5228) and 3.1 weight-% of magnesite (MgCO_3_, 04-009-2317). As for the calcined HYD, the main phase was dolomite (85.4 weight-%), and the other components were huntite (6.7 weight-%), magnesite (6.3 weight-%), calcite (1.4 weight-%), and magnesium oxide (0.2 weight-%).

Calcination caused the decomposition of carbonate phases in the BRU and HYD, but the actual purpose of calcination was to remove the interlayer water molecules in these LDHs to render them effective adsorbents (Lee et al. 2018a).

**Fig. 2:**
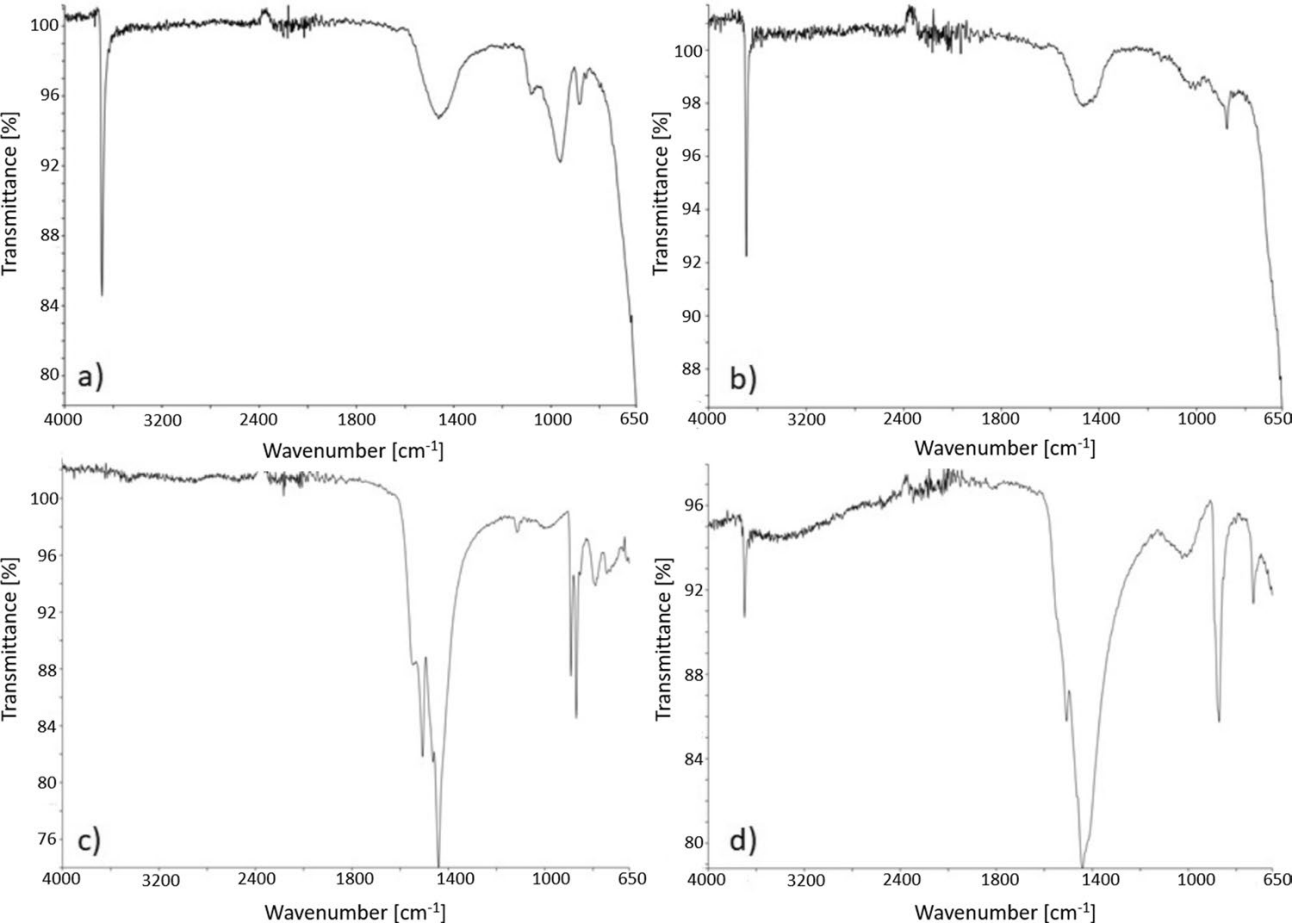
FTIR spectra of **a** non-calcined BRU, **b** calcined BRU, **c** non-calcined HYD, and **d** calcined HYD

The FTIR spectra of the non-calcined and calcined BRU and HYD are shown in Fig. 2. In both the calcined and non-calcined adsorbents, a sharp and strong peak was observed at around 3700 cm^−1^, which is attributed to the structural hydroxyl group (–OH), to the stretching vibration peak, and to the water molecules in the interlayer region (Baliarsingh et al. 2013; Chowdhury et al. 2018; Focke et al. 2014; Lu et al. 2015). In the calcined BRU, the intensity of the peak at around 3700 cm^−1^ decreased due to the decomposition of Mg(OH)_2_ to MgO during calcination (Chowdhury et al. 2018). The band at around 1500 cm^−1^ may be attributed to the carbonate and/or bending vibration of the physisorbed water (Baliarsingh et al. 2013; Chowdhury et al. 2018; Lu et al. 2015). The bands observed in the low frequency region (<1000 cm^−1^) are attributed to the lattice vibration modes of the metal–oxygen and metal–hydrogen bonds (Baliarsingh et al. 2013; Chowdhury et al. 2018; Lu et al. 2015).

Calcined BRU and HYD were selected for adsorption experiments, and for the sake of brevity, they are referred to as BRU and HYD in the subsequent sections.

### Effect of initial pH

**Fig. 3:**
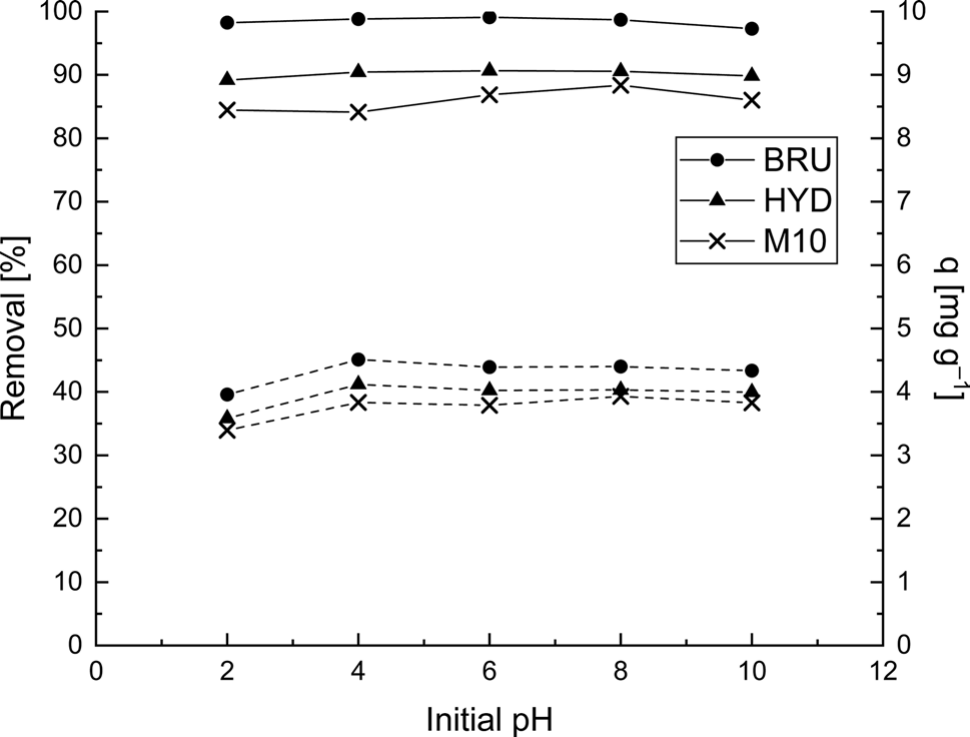
Effect of initial pH on the adsorption of Sb onto the BRU, HYD, and M10. The Sb removal efficiencies (*R%*) are indicated by a solid line, and the adsorption capacities (*q*) are indicated by a dashed line. C_0_: 25 mg L^−1^, adsorbent dosage: 5 g L^−1^, contact time: 24 h, and T: 22–23 °C

pH is one of the most important parameters affecting the adsorption of metal ions in an aqueous solution. Among the dissolved Sb(III) species, the cationic form $\mathrm{Sb}{\left(\mathrm{OH}\right)}_2^{+}$ is the predominant species at pH <1.4. The electrically neutral Sb(OH)_3_ is the principal species at pH 2–11, whereas $\mathrm{Sb}{\left(\mathrm{OH}\right)}_4^{-}$ is the dominant species at pH >11.8 (Kang et al. 2000; Wu et al. 2012). However, when Sb(III), which was used to prepare the model wastewater, is oxidized into Sb(V), a soluble Sb(OH)_6_^−^ species is present at pH >3. The effect of the initial pH ranging from 2 to 10 on the Sb desorption by the BRU, HYD, and M10 was investigated (Fig. 3). The results showed that the removal efficiency and adsorption capacity remained nearly constant at the initial pH of 4–10. At pH 2, the adsorption capacities were slightly lower than those at initial pH values of 4, 6, and 8. This phenomenon may have been caused by the inability of the positively charged Sb(OH)_2_^+^ to enter the positively charged interlayers of the LDHs. Also, the Sb removal at pH 2 may have been influenced by other mineral phases present in adsorbents with negative surface charges. One possible explanation for the nearly constant removal efficiency at different initial pH values is the oxidation of Sb(III) to Sb(V) in the solution. Consequently, the soluble Sb(OH)_6_^−^ species started to dominate in the solution at pH >3, resulting in the improved adsorption efficiency. In addition, the adsorbents were alkaline materials, and the pH increased during the adsorption experiment. Thus, it is possible that apart from Sb(OH)_6_^−^, Sb${\left(\mathrm{OH}\right)}_4^{-}$ species were also present in the solution, enhancing the removal efficiency. The results showed that the BRU and HYD were more effective adsorbents than the M10. When the initial pH was varied from 2 to 10, the removal efficiencies and capacities were 97.3–99.1% and 3.96–4.51 mg g^−1^, respectively. For the HYD and M10, the removal efficiencies varied only slightly, that is, 89.2–90.7% (3.6–4.1 mg g^−1^) and 71.8–88.4% (3.2–3.9 mg g^−1^), respectively. Given that the removal efficiencies and adsorption capacities for Sb were nearly the same at pH 2–10, the initial pH was adjusted to pH 4 in the subsequent experiments.

### Effect of adsorbent dosage

**Fig. 4:**
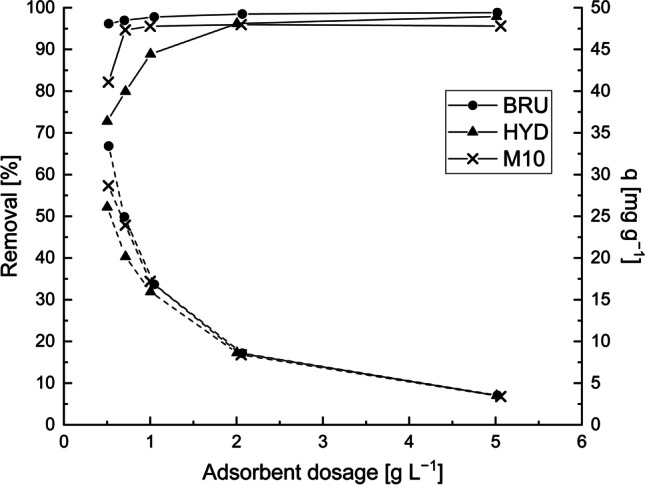
Effect of adsorbent dosage on Sb removal by the BRU, HYD, and M10 from the model solution. The Sb removal efficiencies (*R%*) are indicated by a solid line, and the adsorption capacities (*q*) are indicated by a dashed line. C_0_: ~18 mg L^−1^, initial pH: 4, contact time: 24 h, and T: 22–23 °C

**Fig. 5:**
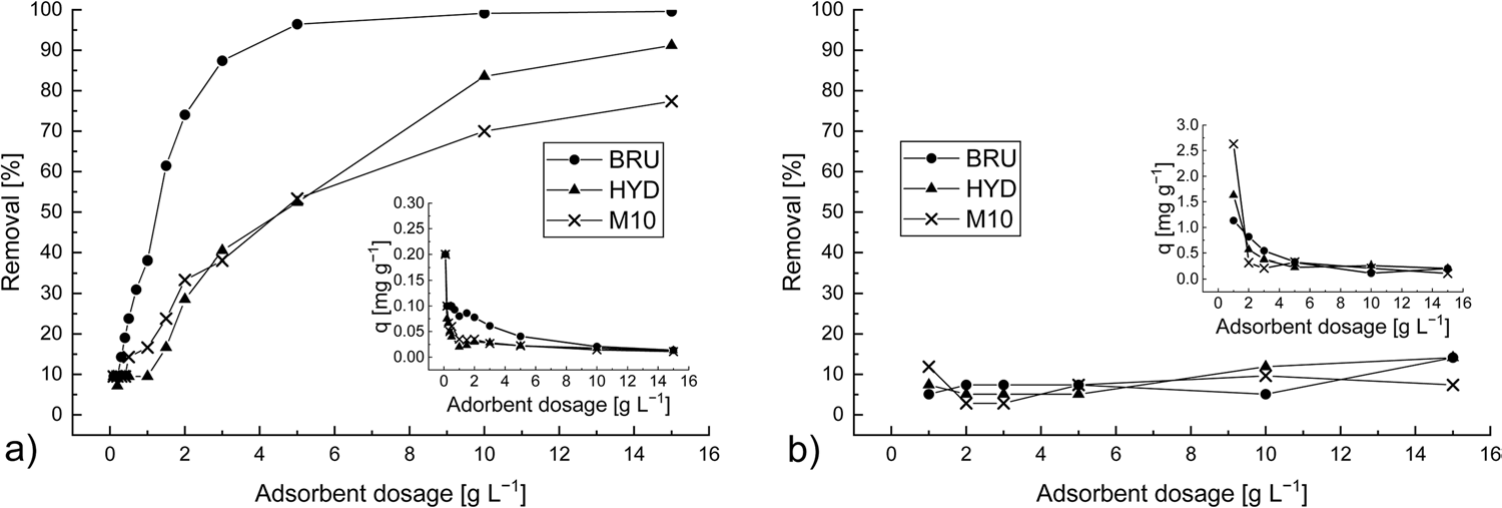
Effect of adsorbent dosage on Sb removal by the BRU, HYD, and M10 from the **a** mine effluent (C_0_: 210 μg L^−1^, pH: 7.18) and **b** textile wastewater (C_0_: 22.13 mg L^−1^, pH: 7.90). In both cases, contact time was 24 h and T was 22–23 °C

The effect of adsorbent dosage on the adsorption of Sb from the model solution and wastewater samples are shown in Figs. 4 and 5. In the model solution (Fig. 4) and mine effluent (Fig. 5a), the removal efficiency increased with increasing adsorbent dosage. A higher adsorbent dosage provides a larger surface area and ultimately more available adsorption sites; consequently, the Sb removal efficiency increases. On the contrary, the adsorption amounts (*q*_*e*_) decreased when the adsorbent dosage increased. This result is due to the increase in the adsorbent-to-adsorbate ratio. An increase in adsorbent dosage under a constant Sb concentration and solution volume leads to the unsaturation of the adsorption sites, resulting in comparatively lower adsorption amount. A similar phenomenon has been observed in other studies (Gaur et al. 2018).

In the model solutions (Fig. 4), the most efficient adsorbent was the BRU. Its removal efficiency was considerably high at 96.2−98.8% for the entire adsorbent dosage range, and its maximum adsorption amount was 33.4 mg g^−1^. The maximum Sb removal efficiencies of the HYD and M10 were approximately 97.9% (*q* = 3.5 mg g^−1^) and 95.9% (*q* = 8.4 mg g^−1^) when their respective dosages were 5 and 2.0 g L^−1^. As shown in Fig. 4, the Sb removal efficiencies of the three adsorbents were considerably high even at low adsorbent dosages. Therefore, 0.5 g L^−1^ was selected as the optimum absorbent dosage.

The removal of Sb ions from the mine effluent and textile wastewater was also studied. In the mine effluent (Fig. 5a), the BRU was also the most efficient adsorbent. Its Sb removal efficiency was approximately 96.5% (*q* = 0.041 mg g^−1^) at a dosage of 5 g L^−1^. Further increases in its dosage did not significantly affect its Sb removal efficiency. As for the HYD and M10, their maximum Sb removal efficiencies were approximately 91.2% (*q* = 0.013 mg g^−1^) and 77.4% (*q* = 0.011 mg g^−1^), respectively, when their dosage was 15 g L^−1^. Meanwhile, the sulfate present in the mine effluent (approximately 1100 mg L^−1^, reported as total S in Table 1) appears to not have competed with the anionic Sb species.

In the textile wastewater (Fig. 5b), the Sb removal efficiency was considerably low (<15%) at adsorbent dosages ranging from 1 g L^−1^ to 15 g L^−1^. Interestingly, a considerable increase in the adsorbent dosage appears to have little effect on the removal efficiency. This phenomenon indicates that some other species present at high concentrations in the textile wastewater matrix competed with the anionic Sb. For instance, the concentration of sulfate was approximately 5500 mg L^−1^ (reported as total S in Table 1). Correspondingly, the removal efficiencies were much lower in the textile wastewater than in the model solution or mine effluent. Previous studies have also reported that divalent anions, such as sulfate, could decrease Sb(V) removal (Lee et al. 2018a; Shan et al. 2014).

### Effect of Sb concentration

**Fig. 6:**
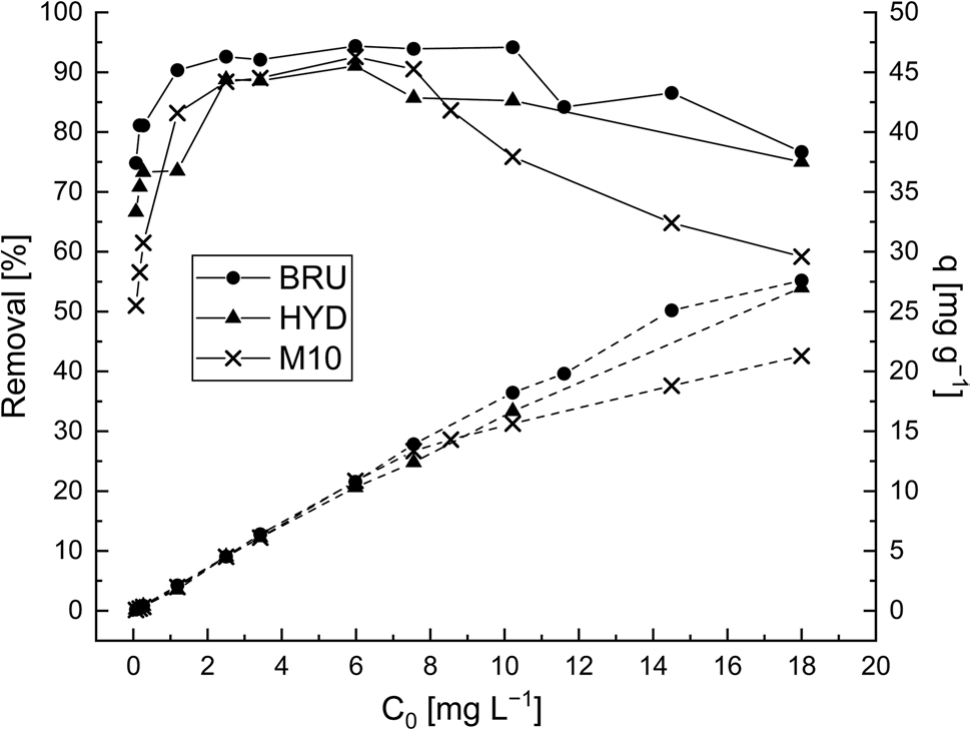
Effect of initial Sb concentration on Sb removal by the BRU, HYD, and M10 from the model solution. The Sb removal efficiencies (*R%*) are indicated by a solid line, and the adsorption capacities (*q*) are indicated by a dashed line. Adsorbent dosage: 0.5 g L^−1^, initial pH: 4, contact time: 24 h, and T: 22–23 °C

Experimental results for the Sb removal by the BRU, HYD, and M10 when the Sb concentration was 0.07–18 mg L^−1^ are shown in Fig. 6. Initially, the Sb removal efficiencies of the adsorbents increased rapidly. This phenomenon can be explained by the fact that the driving force of the concentration gradient increased as the initial Sb concentration increased. When the initial Sb concentration was >6.0 mg L^−1^, the increase in removal efficiencies slowed down; the removal efficiency eventually plateaued and then adsorption started to decrease. Nevertheless, the removal efficiencies in the synthetic wastewater samples were still higher than those in the textile wastewater (Fig. 5b), which supports the view that competition between adsorbates decreased the removal efficiency in the textile wastewater. The trend shown in Fig. 6 can be explained by the fact that at low initial concentrations, the available pores on the adsorbents’ surface could adsorb most of the Sb ions. Thus, the adsorption efficiency increased to a certain level. With the increasing initial concentration, the adsorption sites became saturated and thus could not adsorb Sb any longer. Several investigations have shown that the removal efficiency and adsorption capacity for Sb or any other impurity is concentration-dependent (Bessaies et al. 2020; Fan et al. 2016; Ge et al. 2015; Ghasemi et al. 2014; Nishad et al. 2014; Thangaraj et al. 2020). The maximum Sb adsorption capacities of the BRU, HYD, and M10 were 27.6, 27.0, and 21.3 mg g^−1^, respectively.

### Adsorption isotherms

The Langmuir, Freundlich, Sips, and RP isotherm parameters and curve fits for Sb adsorption were analyzed. The isotherm parameters, correlation coefficients, and *RMSE* values are given in Table 4. Fig. 6 presents the results of the isotherm modelling of the experimental data.

**Table 4:** Parameters of the adsorption isotherms

Experimental/model	Constant/unit	BRU	HYD	M10
Experimental	*q*_*m,exp*_ [mg g^−1^]	27.6	27.0	21.3
Langmuir	*q*_*m,calc*_ [mg g^−1^]	32.513	41.107	22.915
	*b*_*L*_ [L mg^−1^]	1.332	0.432	1.165
	*R*^*2*^	0.956	0.973	0.943
	*RMSE*	2.207	1.528	1.924
Freundlich	*n*_*F*_	2.107	1.613	2.387
	*K*_*F*_ [(mg g^− 1^)(mg L^− 1^)^− 1/n^ ]	15.600	11.098	9.972
	*R*^*2*^	0.891	0.949	0.863
	*RMSE*	3.462	2.106	2.999
Sips	*q*_*m*_ [mg g^−1^]	25.496	34.970	18.936
	*b*_*S*_ [L mg^−1^]	2.400	0.620	1.972
	*n*_*S*_	1.887	1.166	1.771
	*R*^*2*^	0.974	0.975	0.960
	*RMSE*	1.784	1.572	1.687
RP	*K*_*R*_ [L g^−1^]	38.250	15.926	24.664
	*a*_*R*_ [L mg^−1^]^-β^	0.986	0.271	0.960
	β	1.137	1.205	1.064
	*R*^*2*^	0.958	0.974	0.944
	*RMSE*	2.262	1.610	2.010

**Fig. 7:**
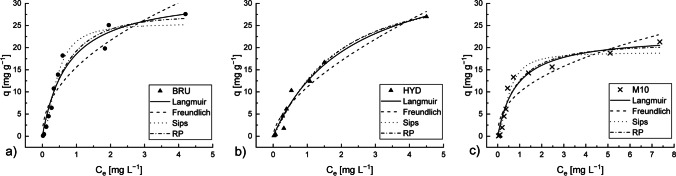
Langmuir, Freundlich, and Redlich–Peterson (RP) isotherm fitting to the Sb adsorption by brucite (BRU), hydromagnesite (HYD), or commercial AQM PalPower M10 adsorbent (M10). Adsorbent dosage: 0.5 g L^−1^, initial pH: 4, contact time: 24 h, and T: 22–23 °C

The experimental data on the Sb adsorption onto the BRU and M10 produced curves with a similar shape. Therefore, both datasets can be represented by the same model. For both adsorbents, the Sips model had the smallest *RMSE* and the highest *R*^*2*^ value. However, the Langmuir and RP were also found to be quite well fit for both adsorbents. In the HYD, three models (Langmuir, Sips, and RP) were an exact fit to the experimental results. Based on the *R*^*2*^ and *RMSE* values, the Freundlich model was less satisfactorily fit to the experimental results (Fig. 7).

Table 5 compares the reported adsorption capacities for different metal oxides, hydroxides, and minerals with those of Sb(III/V). The variations in these values are tremendous, with the smallest value being 0.50 mg g^−1^ and the highest being 214 mg g^−1^. Apart from the *q* values, the initial concentrations and adsorbent dosages varied in the considered studies. For this reason, the comparison is not straightforward. For example, in the study of Xu et al. (2011), a considerably small amount of adsorbent was used to remove a large concentration of Sb. Comparing the capacities of the adsorbents investigated herein with the reported results obtained under similar conditions, we can conclude that our adsorbents have demonstrated an approximately average performance.

**Table 5:** Comparison of the adsorption capacity *q* (mg g^−1^) of various adsorbents for the removal of Sb(III/V) from an aqueous phase. The listed adsorbents are metal oxides, hydroxides, or minerals, and the experiments were conducted at nearly room temperature (20−30**°**C)

Adsorbent	Capacity q [mg g^−1^]	Initial pH	C_0_ [mg L^−1^]	Adsorbent dosage [g L^−1^]	T [°C]	Time [h]	Sb oxid. state	Ref.
Zn-Al Sulfate LDH	210−246^c^	~5.1−5.4	243.52−1217.6 (2−10 mmol)	0.625, 1.24	-	24	V	Ardau et al. 2016
Fe^2+^ -doped Mg-Al layered double hydroxides	231.4^a^	-	36.5−73.0 (0.3-0.6 mmol/L)	10	30	24	V	Kameda et al. 2015
Fe-Mn binary oxide	214^a^	3	24−244	0.0004	20	24	III	Xu et al. 2011
Magnetic nanoparticles supported calcined layered double oxide (MLDO)	180.96^c^	7	10−500	0.2	25	24	V	Lee et al. 2018b
Zn-Fe-LDH	122.03^b^	7	2−100	0.2	20	-	V	Lu et al. 2015
FeOOH	101^a^	3	24−244	0.0004	20	24	III	Xu et al. 2011
MnO_2_	98.6^a^	3	24−224	0.0004	20	24	III	Xu et al. 2011
Synthetic MnOOH	95.5^a^	3	0.5−98	0.4	25	24	V	Wang et al. 2012
Fe-Zr binary oxide	60.4^a^	7	0−25	0.2	25	24	V	Li et al. 2012
ZrO_2_	55.0^a^	7	0−25	0.2	25	24	V	Li et al. 2012
Hematite modified magnetic nanoparticles	36.7^a^	4.1	1−20	0.1	25	36	III	Shan et al. 2014
Diatomite	35.2^a^	6	10−400	4	20	0.5	III	Sarı et al. 2010
Calcined brucite	27.6^c^	4	13	0.5		24	III	Current study
Calcined hydromagnesite	27.0^c^	4	13	0.5		24	III	Current study
AQM PalPower M10	21.3^c^	4	13	0.5		24	III	Current study
FeOOH	18.5^a^	7	0−25	0.2	25	24	V	Li et al. 2012
Bentonite	0.56^a^	6	0.05−4	25	25	24	III	Xi et al. 2011
Bentonite	0.50^a^	6	0.05−4	25	25	24	V	Xi et al. 2011

^a^Langmuir maximum adsorption capacity, *q*_*m.calc*_; ^b^SIPS, *q*_*m.calc*_; ^c^experimental maximum adsorption capacity, *q*_*m.exp*_

### Effect of contact time

**Fig. 8:**
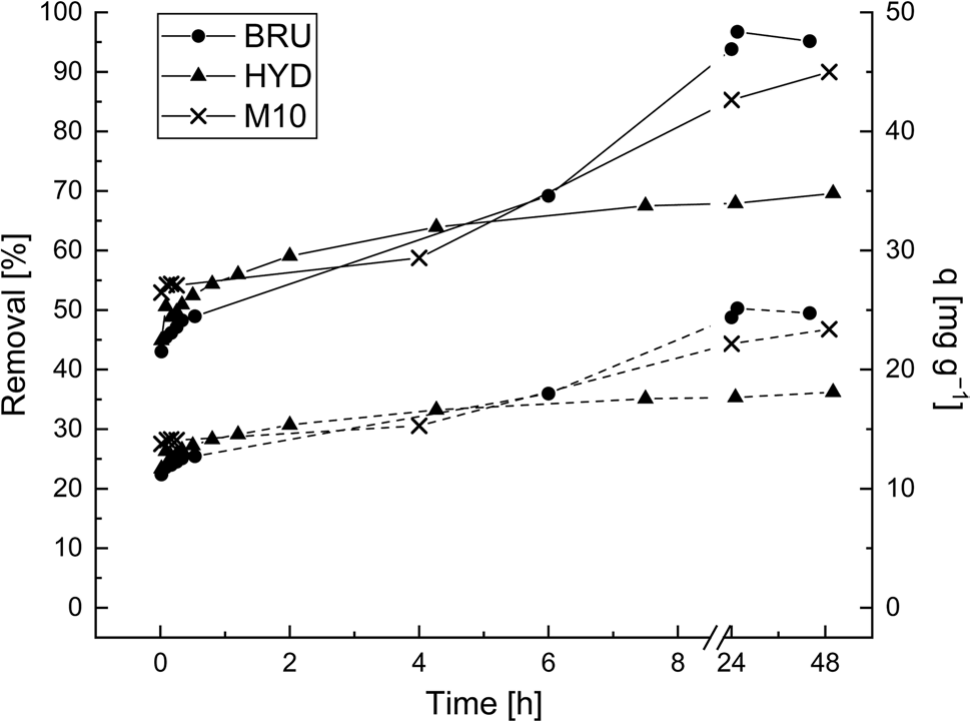
Effect of contact time on Sb removal from the model solution by the BRU, HYD, and M10. The Sb removal efficiencies (*R%*) are indicated by a solid line, and the adsorption capacities (*q*) are indicated by a dashed line. Adsorbent dosage: 0.5 g L^−1^, C_0_: 13 mg L^−1^, initial pH: 4, and T: 22−23 °C

**Fig. 9:**
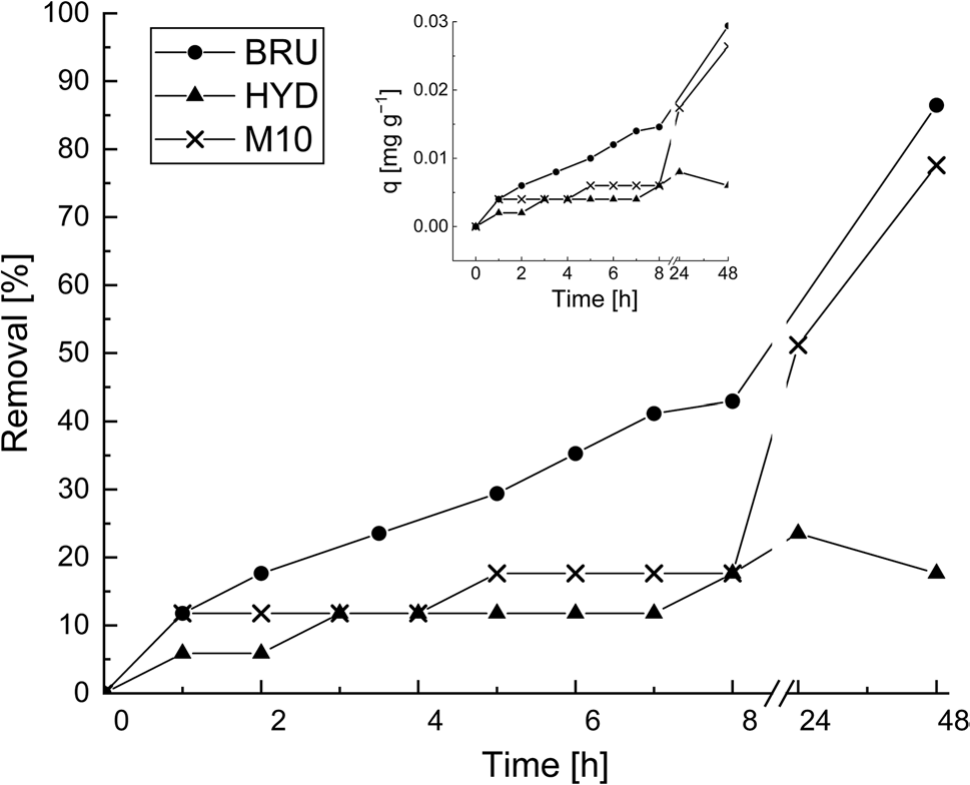
Effect of contact time on Sb removal from the mine effluent by the BRU, HYD, and M10. Adsorbent dosage: 5 g L^−1^, C_0_: 200 μg L^−1^, initial pH: 7.18, and T: 22−23 °C

The kinetic studies involving the BRU, HYD, and M10 were performed at room temperature using the model solution and mine effluent in which the Sb concentrations were 13 and 200 μg L^−1^, respectively. Experiments were not performed for the textile wastewater samples because their Sb removal efficiencies were considerably low, as seen in the earlier experiments. As shown in Figs. 8 and 9, the Sb removal increased with increasing contact time. In the case of the model solution (Fig. 8), an approximately 50% Sb removal efficiency by the BRU, HYD, and M10 was attained after 32, 5, and 1 min, respectively. The contact time required to reach the equilibrium was 25.5 h (96%, 25.0 mg g^−1^) for the BRU and 7.5 h (68.4%, 17.8 mg g^−1^) for the HYD. As for the M10, the maximum removal efficiencies of 85.3% (22.2 mg g^−1^) and 90.0% (23.4 mg g^−1^) were obtained after 24 and 49 h, respectively.

For the mine effluent (Fig. 9), the highest removal efficiency was attained at 48 h; the removal efficiencies were 86.5% (0.03 mg g^−1^) and 77.6% (0.03 mg g^−1^) for the BRU and M10, respectively. For the HYD, the highest removal efficiency of 17.6% (0.06 mg g^−1^) was attained at 24 h, after which the removal efficiency decreased slightly.

### Kinetic modelling

**Fig. 10:**
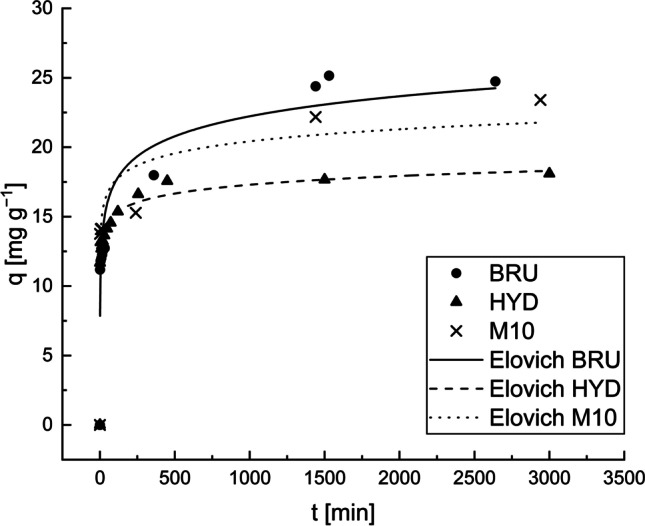
Pseudo-first-order, pseudo-second-order, and Elovich model plots. Adsorbent dosage: 0.5 g L^−1^, C_0_: 13 mg L^−1^, initial pH: 4, and T: 22−23 °C

The pseudo-first-order, pseudo-second-order, and Elovich models were applied to the experimental data. The kinetic parameters, correlation coefficients, and *RMSE* values are presented in Table 6. The Elovich model was clearly the best fit model for the three adsorbents based on the *R*^*2*^ and *RMSE* values. The Elovich model fits the experimental results for the studied adsorbents, as shown in Fig. 10.

**Table 6:** Pseudo-first-order, pseudo-second-order, and Elovich model parameters for the Sb removal by the BRU, HYD, and M10

Experimental/model	Constant [unit]	BRU	HYD	M10
Experimental	*q*_*m(exp)*_ [mg g^−1^]	27.7	18.1	23.4
Pseudo-first-order	*q*_*e(cal)*_ [mg g^−1^]	17.125	14.125	17.198
	*k*_*1*_ [min^−1^]	0.973	1.508	1.608
	*R*^*2*^	0.488	0.825	0.727
	*RMSE*	5.708	1.933	3.992
Pseudo-second-order	*q*_*e(cal)*_ [mg g^−1^]	22.339	15.361	17.970
	*k*_*2*_ [g mg^−1^ min^−1^]	0.005	0.136	0.108
	R^2^	0.717	0.858	0.761
	RMSE	4.244	1.742	3.730
Elovich	*β* [g mg^−1^]	0.481	1.099	0.800
	*υ*_0_ [mg g^−1^ min^−1^]	90.806	165504.000	15463.803
	*R*^*2*^	0.944	0.986	0.940
	*RMSE*	1.888	0.537	1.871

### Weber–Morris intra-particle diffusion model

**Fig. 11:**
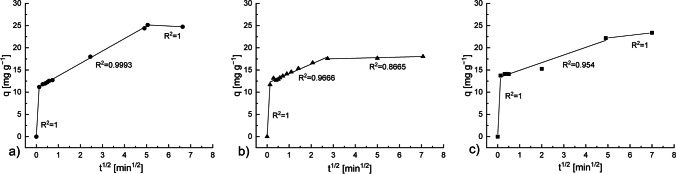
Weber–Morris plots for intra-particle diffusion of the Sb removal on the **a** BRU, **b** HYD, and **c** M10. Adsorbent dosage: 0.5 g L^−1^, C_0_: 13 mg L^−1^, initial pH: 4, and T: 22−23 °C

The plot *q*_*t*_ versus *t*^1/2^ for the Sb adsorption onto the BRU, HYD, and M10 is shown in Fig. 11. When a straight line passes through the origin, it can be assumed that the rate-limiting step in the mechanism is the intra-particle diffusion. As can be seen in Fig. 11, the adsorption of Sb onto the BRU, HYD, and M10 involved three steps, indicating that three mechanisms govern the adsorption. The first stage (from 0 min to 1 min) is the instantaneous or external surface adsorption, wherein Sb is diffused through the solution to the external surface of the adsorbent. The second stage is the gradual adsorption phase, wherein intra-particle diffusion (pore diffusion) is the rate-limiting step. The third stage is the final equilibrium phase, where intra-particle diffusion starts to slow down due to the extremely low concentration of the remaining Sb in the solution (Dong et al. 2015; Salam and Mohamed 2013; Xiong et al. 2020).

### Regeneration of spent adsorbents

Desorption experiments involving the Sb-loaded BRU, HYD, and M10 adsorbents were conducted using deionized water, 1 M NaOH, and 1 M HCl. In the case of deionized water, Sb was not detected in the eluent (Table 7). This finding suggested that the Sb-loaded BRU, HYD, and M10 are stable in water at neutral pH and that Sb is not released back into the water. When 1 M NaOH solution was used as desorption agent, the desorption efficiencies were 28.2%, 39.5%, and 12.9% for the BRU, HYD, and M10, respectively. High desorption efficiencies of approximately 98.6%, 98.7%, and 88.7% for the BRU, HYD, and M10, respectively, were obtained in 1 M HCl solution. These high desorption efficiencies may have been facilitated by the formation of chloro-complexes (Schweitzer and Pesterfield 2010). These results suggested that the spent adsorbents can be regenerated for subsequent use, improving their cost effectiveness, and reducing the operational costs in their application in water treatments.

**Table 7:** Sb desorption using deionized water, 1 M NaOH, and 1 M HCl

Adsorbent	Adsorption		Desorption					
			Deionized water		1 M NaOH		1 M HCl	
	Capacity [mg g^−1^]	Efficiency [%]	Amount of Sb released [mg g^−1^]	Efficiency [%]	Amount of Sb released [mg g^−1^]	Efficiency [%]	Amount of Sb released [mg g^−1^]	Efficiency [%]
BRU	2.84	71.0	0.06	2.1	0.80	28.2	2.80	98.6
HYD	3.04	76.0	0.10	3.3	1.20	39.5	3.00	98.7
M10	2.48	62.0	0.08	3.2	0.32	12.9	2.20	88.7

### Column studies

**Fig. 12:**
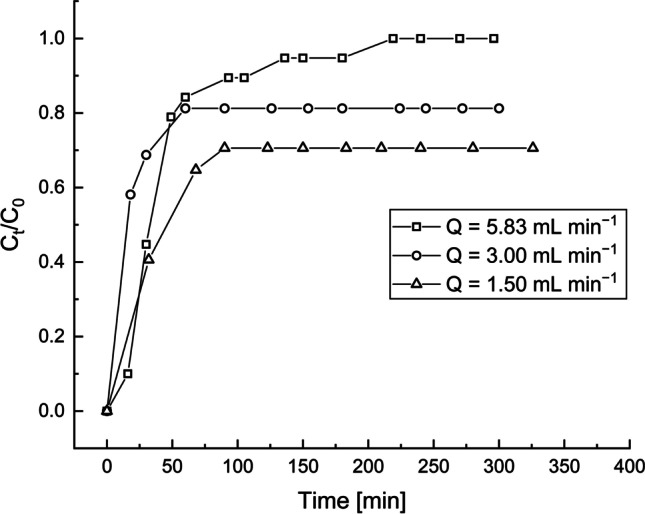
Experimental breakthrough curves for the adsorption of Sb from the mine effluent onto the BRU at three different flow rates. Adsorbent dosage: 100 g, C_0_: 160−190 μg L^−1^, initial pH: 7.18, and T: 22−23 °C

The BRU was the most efficient adsorbent based on the results of the batch adsorption experiments. Thus, the BRU was selected for the column experiments in which mining wastewater (containing ~170 μg L^−1^ Sb) was used. The flow rates of 1.50, 3.00, and 5.83 mL min^−1^ were used to obtain the adsorption breakthrough curves (Fig. 12). At 1.50, 3.00, and 5.83 mL min^−1^ flow rates, the breakthrough times were 3.91, 1.71, and 8.17 min, respectively. The exhaustion time (~183 min) was reached only under the highest flow rate (5.83 mL min^−1^). As for the two other flow rates (1.50 and 3.00 mL min^−1^), a plateau was achieved at 90 and 60 min, respectively. Because the exhaustion time was not achieved under these flow rates, the adsorption capacity for comparative purposes was calculated after the contact time of ~300 min for each flow rate had elapsed. The cumulative amounts of adsorbed Sb were 0.274, 0.297, and 0.405 μg g^−1^ when the flow rates were 1.50, 3.00, and 5.83 mL min^−1^, respectively.

### Mechanistic considerations

The adsorption mechanisms of LDHs have been scrutinized in many earlier studies, and thus we provide a brief summary of those in this section. LDHs are able to remove effectively anionic contaminants such as arsenic (Wang et al. 2018), phosphate (Keyikoglua et al. 2022), chromium (Tran et al. 2019), and antimony (Ungureanu et al. 2015) from water based on the ion exchange of the interlayer anions e.g. CO_3_^2-^ (interlayer ion exchange). In addition, the calcination treatment of LDHs, which was conducted in the present study, enhances the removal efficiency of anionic contaminants by eliminating of water molecules from the interlayer space and also by affecting the interlayer space dimensions (Elhalil et al. 2018; Lee et al. 2018a). This mechanism is referred to as the “memory effect.” In addition, antimony removal can be based on surface adsorption, which can be physical or chemical. In the case of LDH materials, chemical adsorption is more common due to the presence of hydroxyl groups on the hydroxide layer on the surface of LDHs, which are able to interact with cationic species by hydrogen-bonding and the complexation effect (Dong et al. 2022).

## Conclusions

Calcined brucite (BRU) and hydromagnesite (HYD) were studied for the Sb removal from synthetic solution and real wastewaters from mining and textile industries. The commercial adsorbent AQM PalPower (M10) was used as reference material. The batch adsorption experiments showed that the BRU was more effective than the HYD and M10 for Sb removal: their experimental maximum adsorption capacities in model solutions were 27.7, 27.0, and 21.3 mg g^-1^, respectively. The isotherm study results indicated that the adsorption data correlated well with the Sips model in the case of the BRU and M10. For the HYD, the Langmuir, Sips, and RP were the best-fitting models. Moreover, the kinetics of the Sb adsorption onto the three adsorbents indicated that ~50% of the adsorption occurred within 30 min followed by a slow evolution up to ~24 h when the equilibrium was reached. The kinetics could be described well with the Elovich model. Desorption studies showed that 1 M HCl can be used to desorb Sb into the waters.

## Data Availability

All processed data supporting the findings of this study is included in this manuscript.
